# Supplementary material for: Blood Stream Microbiota Dysbiosis Establishing New Research Standards in Cardio-Metabolic Diseases, A Meta-Analysis Study
Source: Microorganisms. 2023 Mar 17;11(3):777. doi: 10.3390/microorganisms11030777 (PMC10052040; doi:10.3390/microorganisms11030777)
Supplement: Supplementary file 1 [file microorganisms-11-00777-s001.zip › microorganisms-2256213-supplementary.pdf]

Table S1. Search strategy for identification of studies to be included in the review

**Search strategy**
#1 (Blood Microbiota OR Blood bacteria a OR Circulating microbiota OR Circulating bacteria OR serum microbial DNA OR Blood microbial signatures OR Circulating microbial signatures)<br>#2 (cardiovascular diseases OR cardiometabolic diseases OR septic shock)<br>#3 (diabetes OR diabetic OR hyperglycemia OR hyperglycaemia OR blood glucose)<br>#4 (#1 AND #2 AND #3)<br>#5 (Addresses[ptyp] OR Autobiography[ptyp] OR Bibliography[ptyp] OR Biography[ptyp] OR pubmed books[filter] OR Case Reports[ptyp] OR Congresses[ptyp] OR Directory[ptyp] OR Duplicate Publication[ptyp] OR Editorial[ptyp] OR Systematic reviews OR Meta-analysis OR Festschrift[ptyp] OR In Vitro[ptyp] OR Lectures [ptyp] OR Legal Cases[ptyp] OR Portraits[ptyp] OR Retracted Publication[ptyp] OR Twin Study[ptyp]<br>#6 (#4 NOT #5)
